# Ambient, Bias‐Free, Green Nanoparticle Synthesis via Microdroplet‐Confined Galvanic Chemistry

**DOI:** 10.1002/smll.74248

**Published:** 2026-06-23

**Authors:** Saptarshi Paul, Andy Berbille, Md Arif Faisal, James H Nguyen, Ashutosh Rana, Zhongxia Shang, Dongho Seo, Ki Min Nam, Jeffrey E. Dick

**Affiliations:** ^1^ Department of Chemistry Purdue University West Lafayette Indiana USA; ^2^ Birck Nanotechnology Center Purdue University West Lafayette Indiana USA; ^3^ Department of Chemistry and Institute for Future Earth Pusan National University Busan South Korea; ^4^ Elmore Family School of Electrical and Computer Engineering Purdue University West Lafayette Indiana USA

**Keywords:** alloy, chemical engineering, electrical conductor, galvanic cell, materials science, nanomaterials, nanoparticle, nanotechnology

## Abstract

Nanoparticles have advanced catalysis, sensing, and biomedical engineering, yet their synthesis often requires complex processes, harsh conditions, or applied electrical bias. Here, we introduce a green, spontaneous, and bias‐free method for the direct deposition of metal nanoparticles on conductive substrates under ambient conditions. In this process, microdroplets containing aqueous metal salts are sprayed onto a conductive substrate templated with zero‐valent Zn microparticles. Within each microdroplet, a confined galvanic exchanged reaction generates Cu, Ag, Pt, and Au nanoparticles with mean diameters below 61 nm. Mechanistic studies indicate that nanoparticle growth arises from the coupling of localized galvanic exchange and lateral charge displacement across the underlying conductor. Unlike recent microdroplet‐based syntheses yielding free‐standing nanoparticles, our method forms substrate‐bound nanoparticles and alloy nanoparticles, without external energy input, non‐aqueous solvents, or external bias, and within minutes. By merging the foundational principles of galvanic chemistry with confined reactivity in microdroplets, these results open a path toward more energy‐efficient and environmentally sustainable synthesis of functional nanomaterials.

## Graphical abstract

Confined galvanic reactions within sprayed microdroplets drive the direct growth of metal nanoparticles on conductive surfaces under ambient conditions. Eliminating the need for external bias and thermal input, this approach offers a simple route toward more sustainable nanoparticle synthesis.

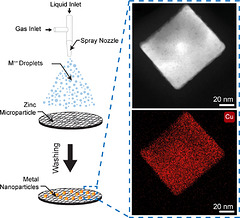

## 1 Introduction

The discovery and manipulation of nanomaterials, particles a billionth of a meter in size, have transformed catalysis [1, 2, 3, 4, 5, 6, 7, 8, 9, 10, 11], sensing [12, 13, 14, 15], environmental remediation [16, 17, 18, 19], and biomedical engineering [20, 21, 22, 23, 24]; largely due to their optical, electronic, and catalytic properties that can be tuned in ways that bulk materials cannot [25]. However, realizing the full potential of nanotechnology depends on the development of scalable and sustainable methods. Established synthesis methods such as physical and chemical vapor deposition (PVD, CVD), sol–gel synthesis, and other thermal or chemical reduction techniques offer good control over morphology and composition. But these processes often require high temperatures, vacuum environments, hazardous reagents, or externally applied energy sources, limiting their cost‐effectiveness, scalability, and sustainability [25]. These constraints point to the need for a synthesis strategy that is simple, ambient, and directly compatible with conductive substrates.

More recently, the emergence of microdroplet‐based synthesis of nanomaterials has introduced an exciting paradigm for accelerated and confined chemical reactions that is beyond their bulk rate. Researchers have demonstrated nanoparticle formation by electrosprayed charged droplets [26, 27, 28], through interfacial reactions between immiscible phases [26], or by colliding aqueous droplets onto electrified surfaces [29, 30, 31]. Yet, many of these methods either require complex setups, or commonly yield aggregates, or free‐standing nanoparticles. The latter case impedes integration into catalytic or energy systems due to poor electrical contact with electrodes. Our previous emulsion‐based electrochemical approach was an attempt to address these issues, but still required externally applied bias and suffered from the limited scalability inherent to the batch emulsion process [32, 33, 34, 35, 36, 37].

Here, we introduce a green, spontaneous, and bias‐free strategy for synthesizing nanoparticles under ambient conditions. We achieve the synthesis of nanoparticles by spraying microdroplets of aqueous metal salt solutions onto a zinc‐templated conductive substrate. The resulting spontaneous redox reactions confined within microdroplets yield discrete substrate‐bond nanoparticles at room temperature and pressure. NanoFarm shows that coupling classical galvanic chemistry with microdroplet confinement provides an energy‐efficient, scalable, and sustainable route to produce nanoparticles.

## 2 Results and Discussion

**FIGURE 1:**
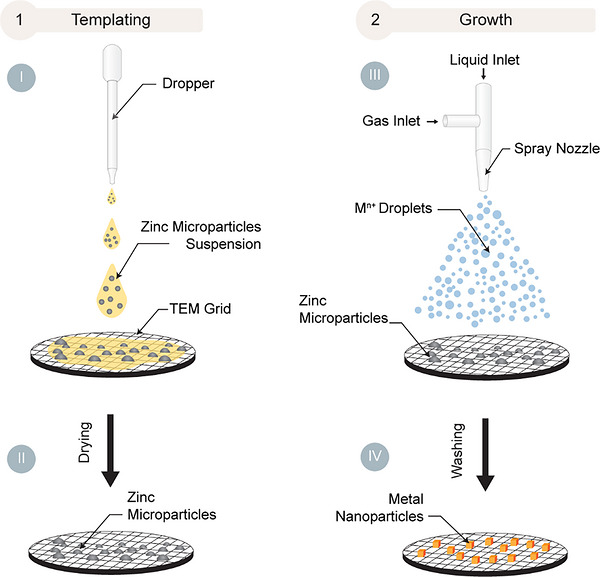
Experimental setup and preliminary demonstration of the nanoparticle synthesis process. Schematic of the droplet‐assisted method for generating metal nanoparticles on a conductive substrate decorated with Zinc microparticles.

Figure 1 illustrates the steps of the herein green and spontaneous method for generating metal nanoparticles under ambient conditions. The process unfolds in two key steps: templating and growth.. In the templating step, represented on the left‐hand side of Figure 1, we decorate a conductive substrate with zinc microparticles. In the case presented, 10 µL of a suspension of Zn microparticles in acetone is drop‐casted onto a TEM grid and allowed to dry completely. Then, we proceed with the growth step during which an aqueous solution of metal precursor salt (50 mm) is sprayed onto the Zn‐decorated substrate using an analytical nebulizer for 2 s, at 135 kPa, and with a tip‐to‐sample distance of 20 cm (see Methods). We selected these parameters after running a series of optimization experiments that we analyzed in Note S1. In these experiments, we varied CuSO_4_ concentration (Figure S1), mass loading of Zn in templating solution (Figure S2), spraying distance (Figure S3), nebulizing gas pressure (Figure S4), duration of spray (Figure S5), and pH of the precursor solution (Figure S6). In the growth step, the microdroplets containing the metal salts collide with the conductor decorated with Zn microparticles and initiate localized redox reactions. Once the spray stops, the substrate is left undisturbed for one minute, allowing the confined reactions within the microdroplets to take place. We propose in a later section that nanoparticle formation occurs through a coupled mechanism involving galvanic exchange and lateral charge displacement across the conductive substrate. Following the reaction, we wash the sample sequentially with deionized water and ethanol to remove residual salts and particulates, then dry it under vacuum. More details about the chemicals and equipment used for this experiment are available in Methods. Finally, we proceed with structural analysis by Scanning Transmission Electron Microscopy–High‐Angle Annular Dark‐Field (STEM‐HAADF) to confirm the synthesis of our metal nanoparticles, which we discuss in the next section.

Inspired by the original experiments of Volta on Zn and Cu piles, we start our study with the synthesis of Cu nanoparticles via the present method. We analyze in detail the Cu nanoparticles produced by this method to then elucidate the underlying electrochemical mechanism and interfacial dynamics that govern spontaneous nanoparticle growth in this method.

**FIGURE 2:**
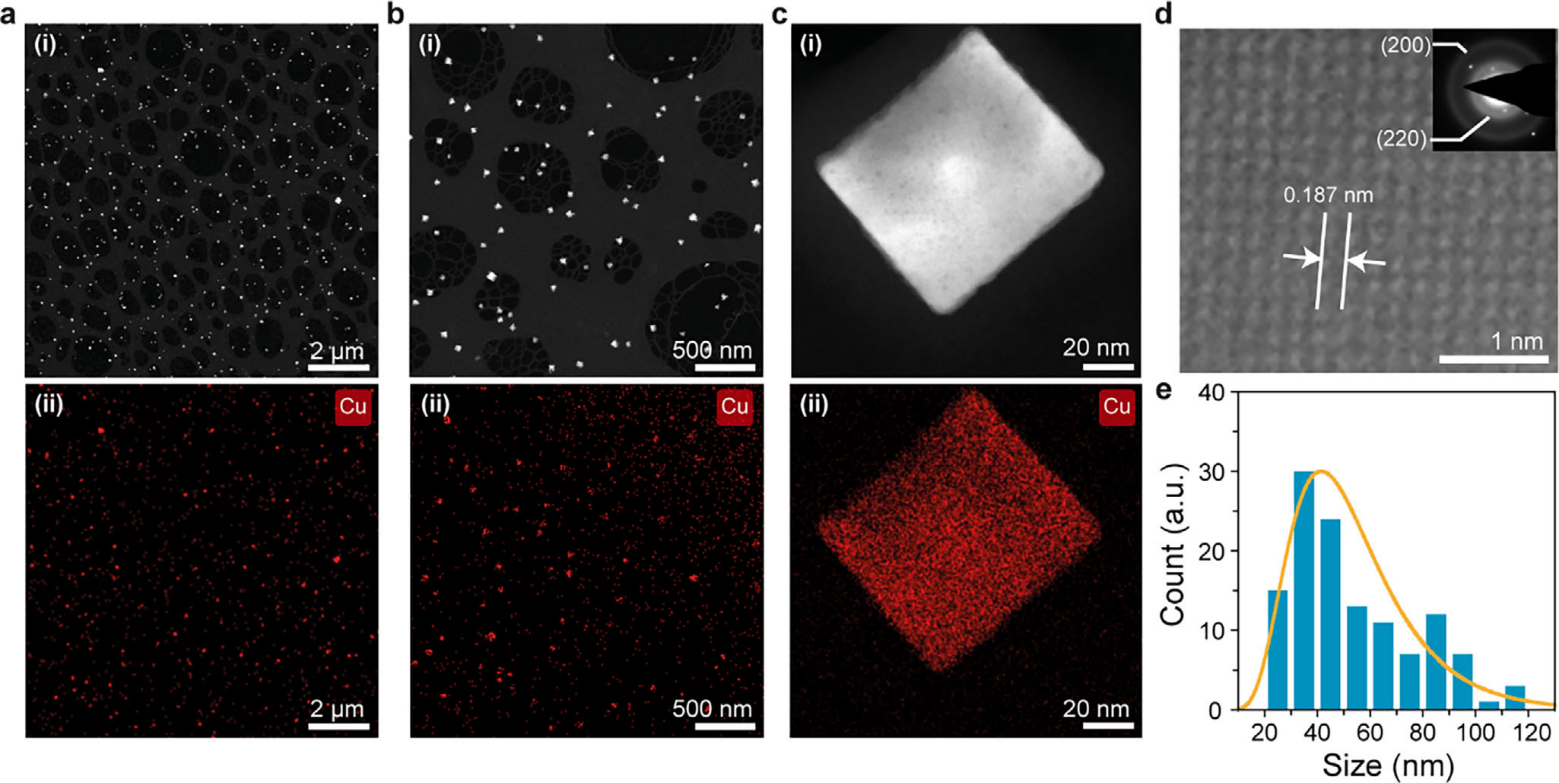
Structural and compositional characterization of copper nanoparticles produced by the herein method. (a) STEM–HAADF (i) image and (ii) corresponding EDX elemental map of copper nanoparticles. (b) STEM–HAADF (i) image and (ii) EDX mapping of copper nanoparticles. (c) STEM–HAADF (i) image and (ii) EDX map of an individual copper nanoparticle. (d) High‐resolution STEM–HAADF image showing the lattice structure of copper nanoparticles, with the SAED pattern shown in the inset. (e) Particle size distribution determined from STEM–HAADF images for a *n* = 123.

We spray a CuSO_4_ solution on a transmission electron microscopy (TEM) grid decorated with Zinc microparticles and examine the Cu nanoparticles synthesized through the herein method using STEM‐HAADF and energy‐dispersive X‐ray spectroscopy (EDX), as illustrated in Figure 2, to evaluate their morphology and composition. As shown in Figure 2a, the TEM grid displayed a uniform distribution of Cu nanoparticles across the entire substrate, formed under ambient conditions. Higher magnification images (Figure 2b) revealed nanoparticles with predominantly cubic morphology, accompanied by a smaller fraction of triangular and globular structures. The cubic shape observed across multiple regions is consistent with previous reports of the formation of metallic copper and copper oxide nanoparticles [38, 39, 40].

To further characterize deposited nanoparticles, a representative Cu‐based nanoparticle, slightly larger than the average to facilitate observations, was analyzed in detail (Figure 2c). The high‐resolution HAADF image (Figure 2d) revealed distinct lattice fringes with an interplanar spacing (d‐spacing) of 0.187 nm, consistent with the (200) plane of metallic zerovalent Cu as observed in the inset selected area electron diffraction (SAED) pattern. This result was confirmed on another particle, as shown in Figure S7. The SAED pattern also exhibits a combination of bright spots and faint concentric diffused rings, revealing the nanoparticles’ polycrystallinity. Elemental mapping (Figure 2; Figure S7) demonstrates that copper is the primary element of the nanoparticle's composition, with some copper nanoparticles comprising oxygen (between 2.1 to 15.9 atomic fraction %) as the experiments are carried out in ambient conditions and Zn contamination (between 0.2 to 8.1 atomic fraction%) possibly due to incomplete galvanic reaction or from contaminations remaining after washing the samples. A statistical analysis (Figure 2e) across more than 100 particles indicated an average particle size of 53.50 ± 22.74 nm, falling well within the typical range for nanoparticles (*d* < 100 nm) [25]. These findings highlight the effectiveness of the present method as a droplet‐mediated approach to generate evenly distributed nanoparticles at room temperature and pressure and indicate most of the product is metallic copper particles.

**FIGURE 3:**
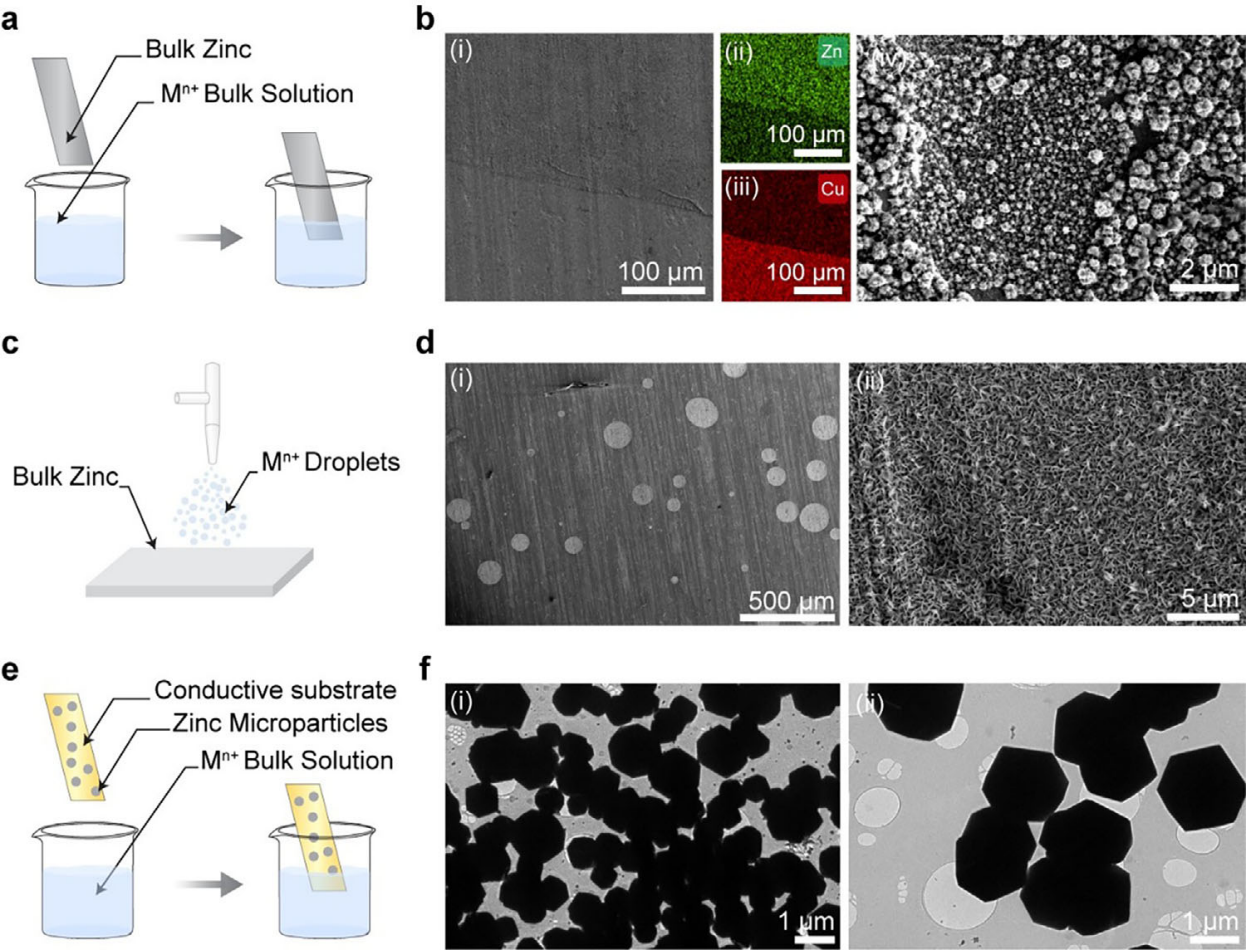
Effects of confinement on galvanically driven nanoparticle formation. (a) Schematic of the bulk Zn–bulk solution configuration, (b) (i) SEM image showing the interface between the portion of bulk zinc immersed in aqueous CuSO_4_ and another section exposed to air. (ii) Zinc and (iii) copper elemental maps of the zinc strip after reaction. (iv) SEM image of the copper‐enriched region. (c) Schematic of the bulk Zn– precursor droplet configuration. (d) SEM images of the site where a nebulized droplet containing CuSO_4_ impacted the zinc surface, at (i) low and (ii) high magnification. (e) Schematic of the Zn microparticle–precursor bulk solution configuration. (f) TEM images of the deposits formed on the conductor under microparticle–bulk conditions, showing (i, ii) two different regions of the TEM grid.

To further understand the parameters governing this spontaneous growth of nanoparticles in the presence of Zn microparticles, and assess the influence of droplet confinement and substrate design on nanoparticle formation, we proceeded to alternatively, or simultaneously, change the size and dimension of the Zn templating agent (microparticles vs bulk) and precursor solution (microdroplet vs bulk). Figure 3 displays the schematic representing each experiment, accompanied by the morphological characterization of the products using either Scanning Electron Microscopy (SEM) or TEM.

In the first case presented in Figure 3a, both zinc and copper were brought into contact in their bulk forms. Thus, a zinc foil immersed (bulk) in a bulk CuSO_4_ solution rapidly developed a black surface layer (Figure S8). SEM images accompanied by EDX mapping (Figure 3b; Figure S9) revealed two distinct regions corresponding to zinc and copper, separated by an interfacial boundary. Higher magnification images showed aggregated, globular macroparticles, indicating uncontrolled nucleation and growth under non‐confined conditions. In the second configuration, we spray the Cu precursor solution on a Zn foil (bulk) (Figure 3c). SEM imaging (Figure 3d; Figure S9) reveals irregularly deposited copper flakes and aggregates distributed over the zinc foil surface. The absence of uniform nanoparticle formation indicates that solely spraying microdroplets is not a sufficient condition to generate the nanoparticles observed in Figures 1 and 2. In the third case, we plunge a conductive TEM grip decorated with Zn microparticles into a bulk Cu precursor solution (Figure 3e). After immersion in CuSO_4_ solution, the resulting product consists of Cu microparticles and aggregates (Figure 3f; Figure S9), with no evidence of the formation of discrete nanoparticles.

Across all three control cases, loss of droplet confinement or asymmetry between micro and bulk form of each component of our system resulted in uncontrolled particle aggregation rather than uniform nanoscale growth. These observations confirm that reaction confinement within microdroplets and use of microscale Zn particles are both essential to form a microreactor that specifically produces the individual nanoparticles observed in Figures 1 and 2.

Finally, to understand the role played by the conductive substrate, we performed complementary in‐operando optical experiments, presented in Note S2 and Figure S10. These experiments show that when we place a macroscopic droplet of CuSO_4_ solution on Zn particles supported on an Indium Titanium Oxide (ITO) substrate, Cu deposition occurs both on and around the Zn surface. However, when we replace the conductive ITO with an insulating glass slide, deposition occurs only onto the Zn particle, emphasizing the role of substrate conductivity in facilitating lateral charge migration. Taken together, these control experiments establish the crucial role played by spatial confinement and the effect of both galvanic exchange and the presence of a conductive substrate in nanoparticle generation. These results aid us in coming with a plausible mechanism, as discussed in detail in the following section.f

**FIGURE 4:**
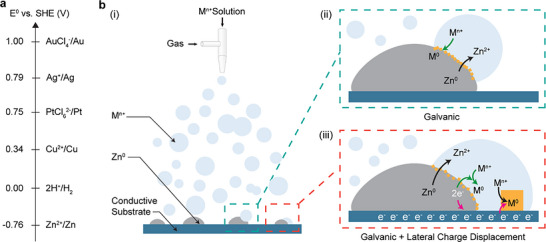
Proposed mechanism for nanoparticle formation via galvanic reactions confined in microdroplets. (a) Standard electrode potential (vs SHE) of the redox couples corresponding to the metals investigated. (b) (i) Schematic illustration of the hypothesized growth mechanism of metal nanoparticles when nebulized microdroplets impact a conductive substrate in the presence of zinc microparticles with (ii) representing the case where the droplet contacts only zinc, leading to localized redox reactions, and (iii) representing the case where the droplet contacts both zinc and the conductive substrate, enabling lateral charge displacement that promotes nanoparticle nucleation and growth away from the zinc particle.

Based on our observations, we propose a plausible mechanism that explains how the present deposition technique spontaneously generates nanoparticles under ambient conditions. A key element is the position of Zn in the electrochemical series relative to more noble metals such as Cu, Pt, Ag, and Au, as shown in Figure 4a. Because the Zn^2^^+^|Zn redox couple has a more negative standard reduction potential among these metals, contact between metallic Zn and a solution of the aforementioned metal salts would act as a galvanic couple, initiating a galvanic displacement, i.e., the dissolved metal ion would be reduced and electrodeposited while Zn gets oxidized to Zn^2^^+^.

In our experiment, as illustrated in Figure 4b(i), microdroplets of precursor metal salt solution (CuSO_4_) land on a conductive substrate that is decorated with Zn microparticles. When a droplet lands on the system, one can identify two configurations. The first case, shown in Figure 4b(ii), is that of a droplet landing directly on Zn microparticles without contacting the conductive substrate below, a classical galvanic reaction occurs. This case would be akin to that of a droplet landing on Zn microparticles sitting onto an insulator and results in a simple galvanic displacement (Figure S10). While the confinement created by the Zn microparticles and the microdroplets can promote nanoparticle formation, most Cu@Zn nanoparticles are likely removed during our washing step due to the weak interaction between most Zn microparticles and the conductive substrate.

On the other hand, when a droplet containing a metal salt collides simultaneously with a Zn microparticle and the conductive substrate underneath, as depicted in Figure 4b(iii), another phenomenon comes into play within the confined volume of the droplet: lateral charge displacement [41, 42]. In this case, electrons generated from the Zn||Cu galvanic couple can laterally migrate across a conductive substrate within the microdroplet, resulting in the reduction of metal ions (Figure S10). As a result, nanoparticles of Cu are electrodeposited away from the Zn particles and strongly bond to the conductive surface even after the surface is washed, as evidenced in Figure 2. It is worth noting that the droplet in this configuration acts as a transient microreactor, enabling rapid nucleation of discrete nanoparticles before neighboring diffusion layers can overlap, thus preventing aggregation [37, 43]. Both mechanisms were further validated in real‐time at the macroscale (Figure S10), confirming that galvanic exchange coupled with lateral charge displacement can drive electrodeposition on and around our templating Zn particle. We also spray our precursor solution on a TEM grid in the absence of Zn microparticles. Nanoparticles were not observed in this case (Figure S11), depicting the importance of the abovementioned galvanic chemistry.

While most observed particles appear as metallic Cu, we also acknowledge the presence of mixed‐phase species, including Cu@Zn alloy structures and minor Cu‐oxides or oxyhydroxide phases, as evidenced in Figure S7. Building on this understanding, we next extend this strategy toward the synthesis of other metals (Ag, Au, and Pt), and the formation that of an alloy of Cu and Pt.

**FIGURE 5:**
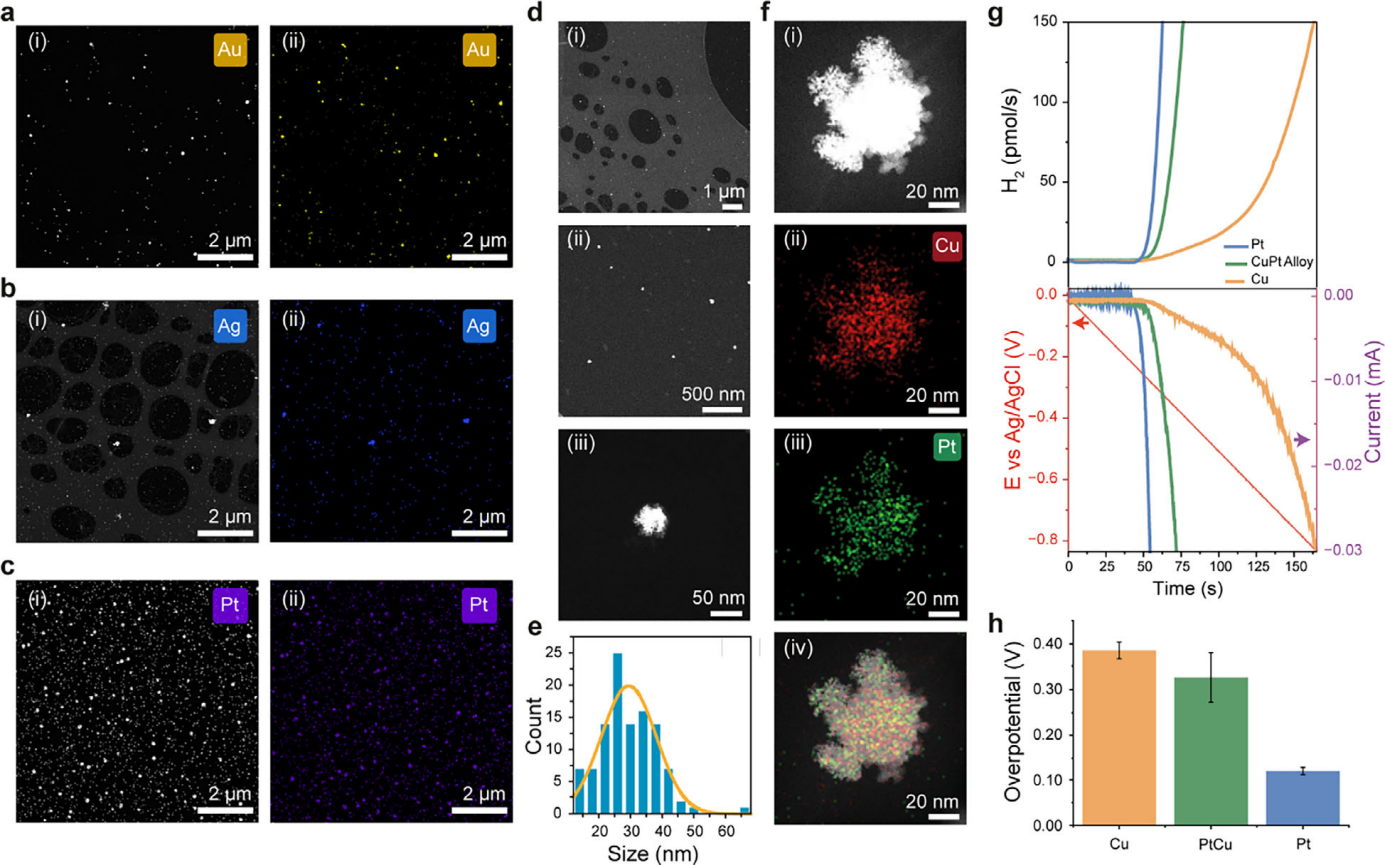
Synthesis of other metallic nanoparticles and alloy nanoparticles by spraying aqueous microdroplets containing metal salt solutions on a substrate templated with zero‐valent Zn microparticles. (i) STEM–HAADF images, and (ii) elemental mapping of particles obtained by spraying a precursor solution containing (a) 50 mm AuCl_4_^−^, (b) 50 mm AgNO_3_, and (c) 50 mm PtCl_6_^2−^. (d) STEM‐HAADF images of particles by spraying a 1:1 molar ratio solution of CuSO_4_ and H_2_PtCl_6_ onto a TEM grid templated with Zn microparticles, shown in (i–iii) by increasing order magnification. (e) size distribution of the particles obtained by spraying an equimolar solution of Cu and Pt precursor on a Zn‐templated TEM grid. (f) (i) High magnification STEM‐HAADF image of the particle, accompanied by the corresponding EDX elemental maps for (ii) Cu, and (iii) Pt as well as (iv) an overlay of the 3 images. (g) Electrochemical Mass‐Spectrometry data for the hydrogen evolution reaction using = Pt, Cu, and CuPt alloy nanoparticles synthesized on glassy carbon. The top represents the hydrogen flow, while the bottom represents the corresponding linear sweep voltammetry data. (h) Overpotentials for the hydrogen evolution reaction using NanoFarmed Pt, Cu, and CuPt alloy synthesized on glassy carbon, acquired using linear sweep voltammetry on a rotating disk electrode. The error bars represent the standard deviation from three independent measurements.

In Figure 5, we first show the results obtained using the herein method to synthesize nanoparticles of Au (Figure 5a), Ag (Figure 5b), and Pt (Figure 5c) from solutions of HAuCl_4_, AgNO_3_, and H_2_PtCl_6_, respectively, sprayed on Zn‐templated TEM grids. The STEM‐HAADF images and EDX elemental map for each of these elements overlap. Size distributions of these particles, presented in Figure S12, show mean diameters of 57.58 ± 19.7 nm, 48.08 ± 16.04 nm, and 60.86 ± 20.51 nm, for Au, Ag, and Pt, respectively. We observe a uniform coverage of particles across different sections of the TEM grid for the 3 elements studied here. Our observations show that the particles’ morphology, varies considerably between metals. Table S1, comparing the nanoparticle mean size with the difference of potential between the standard potential for the reduction of zinc and that of the metals synthesized, shows no correlation between these two parameters. This observation suggests that, although the electrochemical series successfully predicts whether spontaneous nanoparticle formation is thermodynamically favorable, the resulting nanoparticle properties are not determined solely by thermodynamic driving force. Instead, nucleation and growth are likely governed by a combination of kinetic factors, including ion transport within the microdroplet, local reactant depletion, droplet evaporation dynamics, and heterogeneous nucleation processes. Similar departures from simple thermodynamic scaling relationships have been reported in previous studies of nanoparticle growth [37, 44].

Finally, in Figure 5d, we present STEM‐HAADF images of particles  syntheized using a precursor solution containing 25 mm of CuSO_4_ and 25 mm of H_2_PtCl_4_ on a TEM grid decorated with Zn microparticles, using the same parameters as the previous experiments (see the experimental setup in Methods). While we still observe uniform nanoparticle coverage across various observation areas on the TEM grids, STEM–HAADF imaging (Figure 5d(i–iii)) revealed the formation of spherical to irregularly shaped nanoparticles with an average diameter of 53.5 ± 22.74 nm (Figure 5e). Elemental mapping by EDX (Figure 5f) confirmed the coexistence of both Cu and Pt within the same particles, with an average composition of approximately 72.45%± 4.57% of Cu and 27.75% ± 4.77% of Pt (Figure S13). Although the morphology was less uniform than for monometallic systems, EDX mapping clearly shows a consistent Cu–Pt co‐localization, indicative of an alloyed composition (Figure 5f(i–iv)).

To further confirm this last result, we have evaluated the behavior of the different catalysts toward the hydrogen evolution reaction (HER) of CuPt nanoparticles and compared it to that of the Pt and Cu nanoparticles synthesized using the method presented here; all three were grown on glassy carbon macroelectrodes. The electrochemical mass spectrometry (EC‐MS) results in Figure 5g, correlates the hydrogen flux with the ramp of potential and the current output for Cu, Pt, and CuPt‐modified electrodes. We observe a faster ramp of the hydrogen flux (top) for a carbon electrode decorated with Pt, compared to PtCu followed by Cu. Moreover, the overlay of the current and voltage (bottom) indicates that the overpotentials of these three catalysts follow the same trend. However, we could not access these overpotential values at 10 mA cm^−2^, due to the noise created by the formation of hydrogen bubbles covering the electrode surface. Therefore, we conducted an additional experiment using a rotating disk electrode setup (Figure S14 and Methods). The results presented in Figure 5h show that the overpotentials of Cu, Pt, and CuPt reached 0.384 ± 0.018 V, 0.119 ± 0.008 V, and 0.325 ± 0.054 V vs Ag|AgCl, respectively. The intermediate catalytic properties obtained for the CuPt nanoparticles in both experiments indicate that we have synthesized an alloy of the two metals.

Through this last set of experiments, we have demonstrated that microconfined galvanic reactions on a conductive substrate can not only grow single‐element nanoparticles, but also alloys, under ambient conditions, in aqueous solution, without an artificially applied bias. This ability to spontaneously generate active nanoparticle alloys demonstrates the potential of NanoFarm as an energy‐efficient platform for designing functional nanomaterials for electrocatalysis and energy conversion.

## 3 Conclusion

In this work, we established a platform for nanoparticle synthesis by merging the spontaneous redox chemistry of classical galvanic systems with the confinement‐driven reactivity in microdroplets. Through this green, bias‐free, and energy‐efficient process, metal nanoparticles can be spontaneously generated under ambient conditions without the need for external potentials, surfactants, or elevated temperatures. This technique produces well‐defined single isolated nanoparticles bond on a conductive substrates, through a coupled effect of galvanic exchange and lateral charge displacement. While the parametric study conducted on copper synthesis demonstrates sensitivity of nanoparticle growth to experimental conditions, future work will focus on developing methods that enable predictive tuning of particle size, morphology, and surface coverage. Finally, we demonstrate here that this method can generate alloyed nanoparticles in ambient conditions, highlighting the universality and versatility of the approach.

## 4 Methods

### 4.1 Materials and Chemical Reagents

Copper sulfate (CuSO_4_, Sigma–Aldrich, 98%), Chlorauric Acid, (HAuCl_4_, Sigma–Aldrich, 99.995% trace metal basis), Chloroplatinic Acid (H_2_PtCl_6_, Sigma–Aldrich, 8% w/v stock solution), Silver Nitrate (AgNO_3_, Sigma–Aldrich, 99%), Sulfuric Acid (H_2_SO_4_, Fischer Scientific, 92.25%), Zinc Powder (d < 10 µ, Sigma‐Aldrich, ≥98%), Indium Titanium Oxide coated glass (Qunguan, Narwhal Technology Co, Ltd.), Polycrystalline zinc foil (MF‐Zn1400L, MTI Corporation), Holey Carbon TEM grid (Ted Pella), Compressed Air (Indiana Oxygen, Quality Grade D), Glassy Carbon Electrode (CHI Instruments).

### 4.2 Instruments

Analytical Nebulizer (K‐Type concentric nebulizer) was purchased t from ThermoScientific. FEI Quanta 3D FEG Dual Beam scanning electron microscopy (SEM) and energy dispersive Xray spectroscopy (EDX) were used to obtain images of the samples and to confirm the elements present. The voltages used during SEM and EDX were 5 and 10 keV, respectively. A FEI Tecnai G2 20 transmission electron Microscope (TEM) at 200 keV was used to obtain the diffraction pattern of single NPs by using selected area diffraction. A ThermoScientific Themis Z Double Aberration Corrected S/TEM instrument was used to obtain the dark field images and the EDX mapping of the S/TEM samples. A Pine Research rotating disk electrode (1600 rpm) was used to account for the electrocatalytic behavior of the deposited sample for the HER. ECMS measurements were made using a SpectroInlets ECMS premium model with a Pfeiffer Prisma Pro mass analyzer (Soborg, Denmark), Aerosol Spectrometer (11D, Grimm, Germany)

### 4.3 Experimental Setup

The templating solution was prepared by 25 mg of Zn microparticles (≤10 µm) in 20 mL of acetone, reaching a mass loading of 1.25 mg mL^−1^. The precursor solutions were prepared by diluting  CuSO_4_, HAuCL_4_, AgNO_3_ or H_2_PtCl_6_ salt in deionized water, at a concentration of 50 mm.

Our experimental process consists of two main steps. In the templating step, we dropcast 10 µL of the Zn templating solution over a Holey Carbon TEM grid. The sample is then exposed to air until completely dry (>1 min). The TEM grid is now templated with Zn microparticles (Figure S15). Thereafter, we spray the metal precursor solution using an analytical nebulizer (normal size distribution of droplets ∼ 3 µm, Figure S16) onto the Zn‐templated TEM grid for 2 seconds. The tip‐to‐sample distance is set at 20 cm distance, and the nebulizing gas (compressed air) pressure at 135 kPa.  After waiting for 1 min for the galvanic reaction to occur between Zn and Cu, we wash the TEM grid subsequently in water and ethanol for 1 min each to get rid of salts and contaminants. Finally we store the samples (TEM grid) under vacuum conditions to prepare it for characterization or application.

### 4.4 Overpotential Measurement

Measurements of overpotential were performed using a rotating disk electrode (RDE, 3000 rpm) setup (WaveVortex AF01WV10, PineResearch), and a linear sweep voltammetry at a scan rate of 50 mV s^−1^ without iR compensation (CHI601E, CHInstrument, Co). Overpotentials were determined at a current density of 10 mA cm^−2^ for glassy carbon electrode on which Cu, PtCu, and Pt nanoparticles were deposited using the process described previously.

### 4.5 Electrochemical Mass Spectroscopy (EC‐MS)

We employed EC‐Lab to control the Biologic potentiostat attached to the EC‐MS, while the software Zilien controlled the EC‐MS instrument. The EC‐MS m/z = 2 signal was calibrated for the detection of H_2_ using an internal standard method with the aid of a polycrystalline platinum disk electrode in 1m perchloric acid electrolyte. To calculate the production of H_2_, we used *n* = 2 electrons per hydrogen molecule evolved and the Faraday constant to convert the charge to mol of H_2_. In this manuscript, volatile species produced during the experiment in the EC‐MS and passing through the gas‐permeable membrane chips were carried away by flowing Argon (1 mL min^−1^).

## Author Contributions

S.P. conceived the original idea. S.P., A.B., and M.A.F. designed and built the experimental setup. S.P., A.B., M.A.F., J.H.N., A.R., and D.S performed experiments, collected and analyzed the data. S.P., A.B., and Z.S. performed the electron microscopy characterizations. J.E.D. supervised the project and assisted in the interpretation of the results. S.P. and A.B. wrote the manuscript. All authors participated in revising the manuscript.

## Conflicts of Interest

The authors J.E.D, S.P. and A.B. have disclosed an IP with Purdue University that protects the process presented in this paper and its application. The remaining authors declare no conflicts of interest.

## Supporting information




**Supporting File**: smll74248‐sup‐0001‐SuppMat.docx.

## Data Availability

The authors declare that the data supporting the findings of this study are available within the paper and its Supplementary Information files. Should any raw data files be needed in another format they are available from the corresponding author upon reasonable request.
