# Supplementary material for: Ambient, Bias‐Free, Green Nanoparticle Synthesis via Microdroplet‐Confined Galvanic Chemistry
Source: Small. 2026 Jun 23;22(46):e74248. doi: 10.1002/smll.74248 (PMC13485035; doi:10.1002/smll.74248)
Supplement: Supplementary file 1 [file SMLL-22-e74248-s001.docx]

**Supplementary Information**

**Ambient, Bias-Free Nanoparticle Synthesis via Droplet-Confined Galvanic Chemistry**

**Saptarshi Paul^1,5^, Andy Berbille^1,5^, Md Arif Faisal^1^, James H Nguyen^1^, Ashutosh Rana^1^, Zhongxia Shang^2^, Dongho Seo^1,3^, and Jeffrey E. Dick^1,4,*^**

^1^ Department of Chemistry, Purdue University, West Lafayette, IN, 47907, USA

^2^ Birck Nanotechnology Center, Purdue University, West Lafayette, IN, 47907, USA

^3^ Department of Chemistry and Institute for Future Earth, Pusan National University, Busan, 46241, South Korea

^4^ Elmore Family School of Electrical and Computer Engineering, Purdue University, West Lafayette, IN, 47907, USA

^5^These authors contributed equally.

Corresponding Authors: Jeffrey E. Dick ([jdick@purdue.edu](mailto:jdick@purdue.edu))

**This document includes:**

Supplementary Note 1 | Optimization of the NanoFarm Process

Supplementary Table S1| Electrochemical Driving Force vs Mean Particle Size

Supplementary Fig. S1| Study of the Effect of CuSO_4_ Concentration

Supplementary Fig. S2 | Study of the Effect of Zn Mass Loading

Supplementary Fig. S3 | Study of the Effect of Tip-Sample Distance

Supplementary Fig. S4 | Study of the Effect of Nebulizing Gas Pressure

Supplementary Fig. S5 | Study of the Effect of Spraying Duration

Supplementary Fig. S6 | Study of the Effect of pH

Supplementary Fig. S7 | Morphology, Structure and Composition of Cu-based NanoFarmed Particles

Supplementary Fig. S8 | Optical Microscopy Experiment Showing the Galvanic Replacement Occuring at the Surface of a Zinc Foil in Contact with a Macroscale Droplet of CuSO_4_

Supplementary Note 2 | Comments on Supplementary Fig. S9-S13

Supplementary Fig. S9 | Additional Images of Control Experiments

Supplementary Fig. S10 | *In-Operando* Experiment Showing the Deposition of Cu on an ITO glass in seeded with a Stack of Zn Microparticles vs on a Simple Glass Slab.

Supplementary Fig. S11 | TEM images of Holey Carbon Grid Sprayed Upon with CuSO_4_ Precursor Solution in Absence of Zn Microparticles

Supplementary Fig. S12 | Particle Size Distribution of NanoFarmed Au, Ag, and Pt particles

Supplementary Fig. S13 | Morphology and Composition of Particles Obtained by NanoFarming an Equimolar CuSO_4_/H_2_PtCl_6_ Precursor Solution

Supplementary Fig. S14 | Overpotential Measurements

Supplementary Fig. S15 | TEM images Zn particle drop-casted on a TEM grid before the spraying process

Supplementary Fig. S16 | Nebulized Droplet Sizes

**Supplementary Note 1 | Optimization of the NanoFarm Process**

To examine the influence of reaction parameters on nanoparticle formation, we systematically varied both chemical and physical conditions in the NanoFarm process. The standard synthesis employs 50 mM CuSO₄ as the metal precursor and 10 µL of a dispersion of Zn microparticles (10 µm) dispersed acetone with a mass loading of 1.25 mg mL^-1^, sprayed at 135 kPa, respecting a tip-to-sample distance of 20 cm.

When the Cu precursor concentration was reduced to 10 mM (Supplementary Fig. S1a), we observe the formation of nanoparticles. However, the coverage is sparce, with vacant regions on the TEM grid. Meanwhile, using a solution of 100 mM CuSO_4_ (Supplementary Fig. S1b) produces large size particles exceeding 100 nm in size.

Similarly, changing the Zn mass loading influences nucleation density: higher Zn mass loading (2.5 mg mL^-1^) yielded larger particles, exceeding 100 nm in diameter (Supplementary Fig. S2a). Meanwhile lower mass loading (0.25 mg mL^-1^) resulted in sparse coverage of nanoparticles across the grid (Supplementary Fig. S2b) .

Physical parameters also played a decisive role. Increasing it to 25 cm led to nanoparticles but reduced particle density significantly (Supplementary Fig. S3a), while decreasing the spray distance to 15 cm led to larger, clustered, and occasionally dendritic morphologies (Supplementary Fig. S3b).

Nebulizing gas pressure influenced droplet focusing at lower pressure (100 kPa), a broader spray cone produced a wide range of particle sizes as seen in the image (Supplementary Fig S4a). whereas higher pressure (170 psi) created over-concentrated jets that promoted aggregation (Supplementary Fig S4b).

Prolonged spray duration also favored dendritic growth and oversized particles (Supplementary Fig. S5).

Finally, the pH of the precursor solution proved critical. Under basic conditions, Cu(OH)_2_ precipitates formed readily, while acidic media led to nanoparticles with poor surface coverage (Supplementary Fig. S6).

| Metal | E°(Mⁿ⁺/M) (V) | ΔE vs Zn (V) | Mean particle size (nm) |
| --- | --- | --- | --- |
| Cu | 0.34 | 1.10 | 53 |
| Pt | 0.75 | 1.51 | 60 |
| Ag | 0.79 | 1.55 | 48 |
| Au | 1.00 | 1.76 | 58 |

**Supplementary Table S1 | Electrochemical driving force vs mean particle size. a-c** Table comparing the difference between the reduction potential of Cu, Pt, Ag, Au reported in the main text, with the mean particle size of the synthesized metals.

**Supplementary Fig. S1 | Study of the Effect of CuSO_4_ Concentration.** **a-c**, TEM images of Cu nanoparticles obtained when Cu salt concentration was 10 mM. Scale bar denotes 1 µm (**a, b**), and 0.5 µm (**c**). **d**, Particle size distribution for 10 mM CuSO_4_ precursor solution. **e-g**, TEM images of Cu nanoparticles obtained when Cu salt concentration was 100 mM. Scale bar denotes 1 µm (**e, f**) and 0.5 µm (**g**). **h,** Particle size distribution for 100 mM CuSO_4_ precursor solution.

**Supplementary Fig. S2 | Study of the Effect of Zn Mass Loading.** **a-b**, TEM images of Cu nanoparticles obtained when Zn particles concentration was 50 mg/20 mL. Scale bar denotes 1 µm. **c,** Corresponding particle size distribution. **d-f,** TEM images of Cu nanoparticles obtained when Zn particles concentration was 5 mg/20 mL. Scale bar denotes 1 µm.

**Supplementary Fig. S3 | Study of the Effect of Tip-Sample Distance.** **a-b,** TEM images of Cu nanoparticles obtained when the distance between the nebulizer and substrate was 25 cm. Scale bar denotes 1 µm. (c) Corresponding particle size distribution. **d-e,** TEM images of Cu nanoparticles obtained when the distance between the nebulizer and substrate was 15 cm. Scale bar denotes 1 µm. **f,** Corresponding particle size distribution.

**Supplementary Fig. S4 | Study of the Effect of Nebulizing Gas Pressure.** **a-b,** TEM images of Cu nanoparticles obtained when the pressure of the nebulizer was 100 kPa. Scale bar denotes 1 µm. **c,** Corresponding particle size distribution. **d-e,** TEM images of Cu nanoparticles obtained when the pressure of the nebulizer was 175 kPa. Scale bar denotes 1 µm. **f,** Corresponding particle size distribution.

**Supplementary Fig. S5 | Study of the Effect of Spraying Duration.** **a-c,** TEM images of Cu nanoparticles obtained with 5 seconds for spraying. Scale bar denotes 1 µm. **d,** Corresponding Size distribution.

**Supplementary Fig. S6 | Study of the Effect of pH.** **a-c,** TEM images of Cu nanoparticles obtained when pH was acidic (2.5). Scale bar denotes 1 µm.

**Supplementary Fig. S7 | Morphology, Structure and Composition of Cu-based NanoFarmed Particles**. a-h, STEM-HAADF and corresponding EDX images of Cu nanoparticles obtained (Cu in blue, O in green, and Zn in cyan) (Scale bar for (a)-(d): 200 nm; (e)-(h): 50 nm). i, STEM image showing a large area of Cu nanoparticles on TEM grid (Scale bar: 2µm) and, j, corresponding atomic fractions of the nanoparticles depicting its elemental composition containing Cu, O and Zn and k, HAADF image of nanoparticles and corresponding lattice fringes and SAED inset (above) of a Cu nanoparticle with EDX mapping of Cu in red in the inset (below).

**Supplementary Fig. S8 | Optical Microscopy Experiment Showing the Galvanic Replacement Occuring at the Surface of a Zinc Foil in Contact with a Macroscale Droplet of CuSO_4_. a,** Image of a Zn foil under galvanic reaction with a CuSO4 droplet and, **b,** corresponding high-resolution real-time optical microscopy probing the galvanic reaction. (Scale bar is 1mm.)

**Supplementary Fig. S9 | Additional Images of Control Experiments.** Representative images showing the morphologies obtained when, **a and b**, both Zn particles and CuSO₄ solution in bulk, **c and d**, Zn in bulk form with CuSO_4_ delivered via microdroplets, and,**e and f,** Zn confined as microparticles with CuSO₄ in bulk.

**Supplementary Note 2 | Comments on Supplementary Fig. S9-S13.**

To gain real-time insight into the mechanism of nanoparticle formation, we performed *in-operando* optical microscopy experiments using Zn macroparticles (d≤10 µm) and individual microdroplets of CuSO_4_ (25 mM) solution (1–5 mm in diameter). When a single droplet was cast onto a Zn foil, the contacted region rapidly turned black, accompanied by visible motion of fine particulates within the droplet, signaturing of an ongoing galvanic reaction between Zn and Cu ions, as shown in Supplementary Fig. S9.

To probe the effect of substrate conductivity and lateral charge displacement, we repeated the experiment using identical Zn macroparticles placed on either a conductive (ITO-coated glass) or insulating (plain glass) substrate. As shown in Supplementary Fig. S10, on the conductive surface, Cu deposition occurred both on and around the Zn particle, indicating lateral migration of electrons through the substrate and reduction of Cu²⁺ at remote sites. In contrast, on the insulating surface, deposition was restricted to the Zn region, consistent with the absence of lateral electron transport.

We also tested the effect of spraying CuSO_4_ solution without Zn being present. As shown Supplementary Fig. S11, we did not observe the presence bond nanoparticles on the TEM grid after washing in this configuration.

We also show in Supplementary Fig. S12 TEM images of the TEM grid templated with Zn microparticles that has not contacted a CuSO_4_ solution to have a better idea of the state of the sample before synthesis.

Finally, we measured the size of the droplet we produced in the optimized nebulization parameters described previously, and we found they display a normal distribution around 5 µm in diameter, as shown in Supplementary Fig. S13.

**Supplementary Fig. S10 | *In-Operando* Experiment Showing the Deposition of Cu on an ITO glass in seeded with a Stack of Zn Microparticles vs on a Simple Glass Slab. a,** Image of a Zn particle undergoing galvanic reaction with a CuSO4 droplet on ITO (conductive surface) and, **b,** Image of a Zn particle undergoing galvanic reaction with a CuSO4 droplet on glass slide (insulating surface).

**Supplementary Fig. S11 | TEM images of Holey Carbon Grid Sprayed Upon with CuSO_4_ Precursor Solution in Absence of Zn Microparticles. a-b,** Image of 2 different portions of a TEM grid on which CuSO_4_ has been sprayed upon in absence of Zn microparticles after washing in water and ethanol.

**Supplementary Fig. S12 | Particle Size Distribution of NanoFarmed Au, Ag, and Pt particles.** Particles size distribution from TEM images for **a,** NanoFarmed Au nanoparticles, **b,** Nanofarmed Ag nanoparticles, **c,** NanoFarmed Pt nanoparticles.

**Supplementary Fig. S13 | Morphology and Composition of Particles Obtained by NanoFarming an Equimolar CuSO_4_/H_2_PtCl_6_ Precursor Solution.** **a,** shows a representative HAADF image depicting CuPt alloy nanoparticles and, **b,** the corresponding EDX ratios. **c,** High Resolution image of a single nanoparticle and its corresponding EDX mapping and, **d,** representing a spectrum of the image showing both Cu and Pt peaks.

**Supplementary Fig. S14 | Overpotential Measurements.** Linear Sweep Voltammetry SV of bare GC (black), and Cu- (red), PtCu- (blue), and Pt (purple)-decorated electrodes for the HER in 1.0 M H_2_SO_4_ at 25 ℃. Measurements were performed using a rotating disk electrode (RDE, 3000 rpm) at a scan rate of 50 mV s^−1^ without iR compensation.

**Supplementary Fig. S15 | TEM images Zn particle drop-casted on a TEM grid before the spraying process. a-b,** Image of 2 different portions of a TEM grid after templating and before spraying the metal salt solution.

**Supplementary Fig. S16 | Nebulized Droplet Sizes.** The left shows the background measured before spraying droplet. The graph on the right shows the size distribution plot of the microdroplets sprayed at 135 kPa and 20 cm of tip-sample distance along with the background
